# Structural Engineering Effects on Hump Characteristics of ZnO/InSnO Heterojunction Thin-Film Transistors

**DOI:** 10.3390/nano12071167

**Published:** 2022-03-31

**Authors:** Qi Li, Junchen Dong, Dedong Han, Dengqin Xu, Jingyi Wang, Yi Wang

**Affiliations:** 1 Institute of Microelectronics, Peking University, Beijing 100871, China; 1901111209@pku.edu.cn (Q.L.); 2001213007@pku.edu.cn (D.X.); 1901213283@pku.edu.cn (J.W.); imewangyi@pku.edu.cn (Y.W.); 2 School of Information & Communication Engineering, Beijing Information Science and Technology University, Beijing 100101, China

**Keywords:** transparent conductive oxides, InSnO, ZnO, thin-film transistors, hump phenomenon, channel structure

## Abstract

Transparent conductive oxides (TCO) have been extensively investigated as channel materials for thin-film transistors (TFTs). In this study, highly transparent and conductive InSnO (ITO) and ZnO films were deposited, and their material properties were studied in detail. Meanwhile, we fabricated ZnO/ITO heterojunction TFTs, and explored the effects of channel structures on the hump characteristics of ZnO/ITO TFTs. We found that V_hump_–V_ON_ was negatively correlated with the thickness of the bottom ZnO layer (10, 20, 30, and 40 nm), while it was positively correlated with the thickness of the top ITO layer (3, 5, 7, and 9 nm), where V_hump_ is the gate voltage corresponding to the occurrence of the hump and V_ON_ is the turn-on voltage. The results demonstrated that carrier transport forms dual current paths through both the ZnO and ITO layers, synthetically determining the hump characteristics of the ZnO/ITO TFTs. Notably, the hump was effectively eliminated by reducing the ITO thickness to no more than 5 nm. Furthermore, the hump characteristics of the ZnO/ITO TFTs under positive gate-bias stress (PBS) were examined. This work broadens the practical application of TCO and provides a promising method for solving the hump phenomenon of oxide TFTs.

## 1. Introduction

Oxide thin-film transistors (TFTs) are one of the most important applications of transparent conductive oxides (TCO). Recently, TFTs based on TCO have been extensively investigated for the purpose of achieving transparent and flexible displays [1,2,3,4]. Among the candidates of TCO, InSnO (ITO), and ZnO are considered to be promising channel materials of TFTs since they combine excellent electrical properties and high transmittance [5,6]. Furthermore, heterojunction TFTs, which have bilayer-structure channel, have currently drawn considerable attention. Compared with the conventional oxide TFTs with single-layer channels, the heterojunction TFTs are more flexible in device configuration; thus, they better balance performance with stability [7,8,9]. Therefore, it is necessary to investigate the heterojunction oxide TFTs further.

As a crucial issue to be solved, the hump phenomenon emerging in transfer curves negatively shifts the turn-on voltage (V_ON_) of the oxide TFTs, which not only adversely affects pixel brightness but also increases the power consumption of the displays [10]. The hump phenomenon has been widely examined for the single-channel oxide TFTs [11,12,13,14]. Mechanisms including edge effects, charge trapping, multiple current paths, and creation of donor/acceptor-like defects have been proposed [15,16,17,18,19,20]. Nevertheless, there are relatively few studies on the hump phenomena of heterojunction TFTs. Maeng et al. reported the appearance of humps in heterojunction TFTs as the total channel thickness exceeded 80 nm, and this was explained as being a result of back channel conduction [21]. Zhao et al. demonstrated that the humps in heterojunction TFTs are related to channel thickness and results from large total carrier numbers [22]. Even though these studies involve the hump phenomenon in heterojunction TFTs, the effects of channel structure on hump characteristics have not yet been fully studied. Therefore, further investigation is required to make an in-depth and comprehensive analysis of the influence of channel structure on hump characteristics.

In this work, we examined ZnO/ITO heterojunction TFTs. A systematic study on the characteristics of transparent conductive ITO and ZnO films was conducted, including optical transmittance, carrier concentration, material components, and surface morphology. Furthermore, we determined the effects of the channel structure on the hump characteristics of ZnO/ITO TFTs, and explored the hump characteristics under positive gate-bias stress (PBS).

## 2. Experimental Methods

### 2.1. Fabrication of Films and Devices

**Figure 1:**
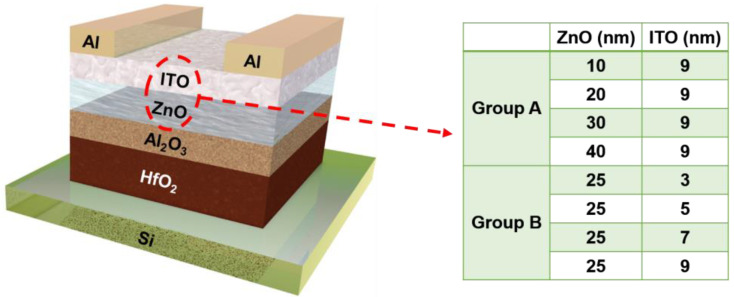
Schematic of the ZnO/ITO TFTs used in this article.

The ZnO and ITO films were deposited on both amorphous glass substrates and single-crystal Si substrates, in which ZnO film was deposited by atomic layer deposition (ALD) at 120 °C and ITO film was sputtered at room temperature. Figure 1 illustrates the device structure of the ZnO/ITO TFTs. A heavy-doped Si wafer was utilized as a substrate and gate electrode. Firstly, 25-nm HfO_2_ was sputtered at room temperature, and 15-nm Al_2_O_3_ was deposited by ALD at 100 °C. Subsequently, the ZnO/ITO heterojunction channel was deposited, where the ZnO layer and ITO layer were deposited using the deposition process mentioned above. Trimethylaluminum (TMA), diethylzinc (DEZ), and deionized water (H_2_O) were the precursors for the aluminum, zinc, and oxygen elements, respectively. Finally, a 100-nm Al film was sputtered as source/drain electrode. Two experimental groups (A and B) were designed, and corresponding channel structures are also shown in Figure 1.

### 2.2. Characterization of Films and Devices

Before measurement, the films and devices were subjected to post-annealing treatment at 100 °C in vacuum for 1 h. To obtain the transparency and optical band gap of the ZnO and ITO films, we tested the transmittance (Zolix Omni-λ500, Beijing, China). The carrier concentration and resistivity were determined by Hall measurement (Ecopia HMS-3000, Anyang, South Korea). X-ray photoelectron spectroscopy (XPS, Kratos Axis Ultra, Manchester, United Kingdom) was performed to analyze the chemical composition and elemental states. Furthermore, the surface morphology and microstructure were characterized by atomic force microscope (AFM, Dimension Icon, Bruker, Billerica, MA, USA; Tipsmodel, Scan ASYST-Air) and transmission electron microscope (TEM, FEI Tecnai F20, Hillsboro, OR, USA), respectively. Furthermore, all the electrical characteristic curves of the TFTs were characterized using a semiconductor device analyzer (Agilent B1500A, Palo Alto, CA, USA) under dark ambient conditions at room temperature.

## 3. Results and Discussion

### 3.1. Material Properties of ZnO and ITO Films

**Figure 2:**
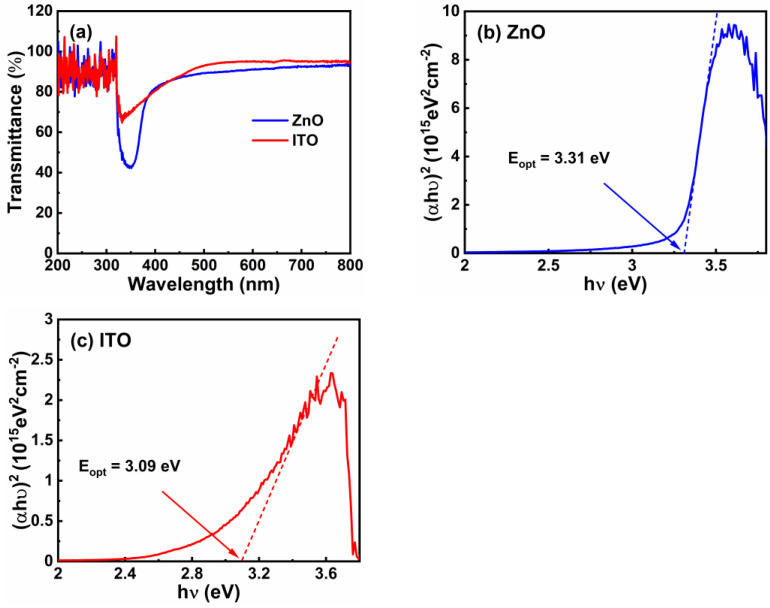
(**a**) Transmittance of ZnO and ITO films. (αhν)^2^-hν curves of (**b**) ZnO and (**c**) ITO films. ZnO and ITO films were deposited on glass substrates.

The transmittance spectra of the ZnO and ITO films on glass substrates were characterized, as revealed in Figure 2a. The testing methods were as follows. First, a bare glass substrate was measured as the reference value (T_r_). Then, the ZnO and ITO films on the glass substrates were measured as the characteristic value (T_c_). Finally, we calculated the transmittance with the characteristic value divided by the reference value (T_r_/T_c_). Clearly, both the ZnO and ITO films possessed an average optical transparency of over 90% in the visible light range. Furthermore, transmittance of the ITO film was higher than that of the ZnO film. The relationship between the optical energy gap (E_opt_) and (αhν)^2^ of the ZnO and ITO films is shown in Figure 2b,c, respectively. Here, the E_opt_ of ZnO and ITO films were calculated using the following equations:

(1) $$\alpha =\frac{1}{t}\ln\frac{1}{T}$$

(2) $${\left(\alpha h\nu \right)}^{2}=D\left(h-E_{\mathrm{opt}}\right)$$

where α is the optical absorption coefficient; t is the thickness of the film; T is the average optical transparency; h is the Planck constant; and D is a constant [23]. It is shown that the E_opt_ value of the ZnO and ITO films is 3.31 and 3.09 eV, respectively.

**Figure 3:**
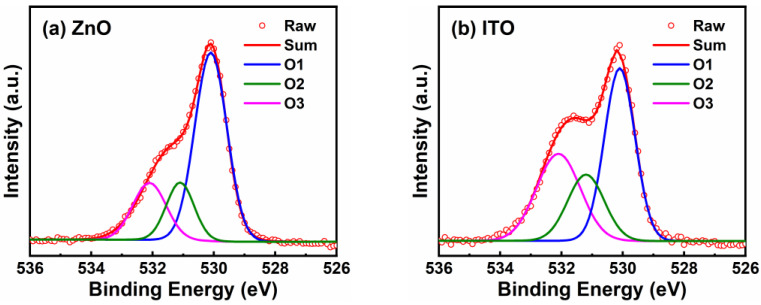
XPS spectra of (**a**) ZnO and (**b**) ITO films. ZnO and ITO films were deposited on Si substrates.

The carrier concentration and conductivity of the single ITO and ZnO films were determined by Hall measurement, as shown in Table 1. The results showed that the carrier concentration and conductivity of the ITO film were three orders of magnitude higher than those of the ZnO film. Figure 3 shows the XPS O 1 s spectra for the ZnO and ITO films. Firstly, the binding energy (BE) was calibrated by the standard C 1 s line at 284.80 eV [24]. Based on Gaussian fitting, the O 1 s spectra were divided into three individual peaks centered at ~530.1 eV (O1 peak), ~531.1 eV (O2 peak), and ~532.1 eV (O3 peak). The O1 peak represents the metal–oxygen lattice whereas the O2 peak represents oxygen vacancy. The O3 peak, however, is usually related to adsorbed oxygen [25,26]. Here, the area ratios of oxygen vacancy in the ZnO and ITO films were calculated to be 16.68% and 21.04%, respectively. Since the oxygen vacancy can serve as a shallow donor, the variation of oxygen vacancy concentration directly alters the carrier concentration of the film [27]. The intensity ratio of the O2 peak is consistent with the Hall effect results.

**Figure 4:**
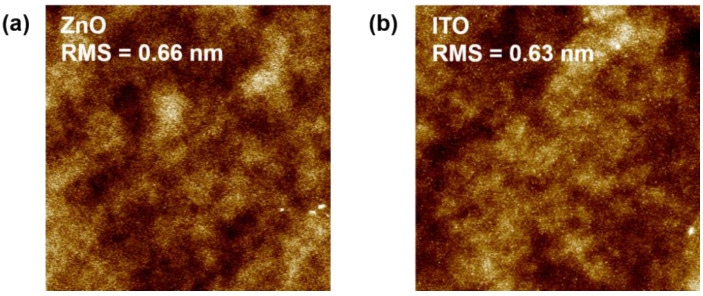
AFM images of (**a**) ZnO and (**b**) ITO films. ZnO and ITO films were deposited on Si substrates.

We also examined the surface morphology of the ZnO and ITO films by means of AFM, as shown in Figure 4. The AFM images were measured in a 5 µm × 5 µm area. The root mean square (RMS) roughness of the ZnO and ITO films was 0.66 and 0.63 nm, respectively. A smooth surface is beneficial to reduce the scattering center and optimize the electrical properties of the oxide TFTs [28].

### 3.2. Hump Characteristics of ZnO/ITO TFTs

**Figure 5:**
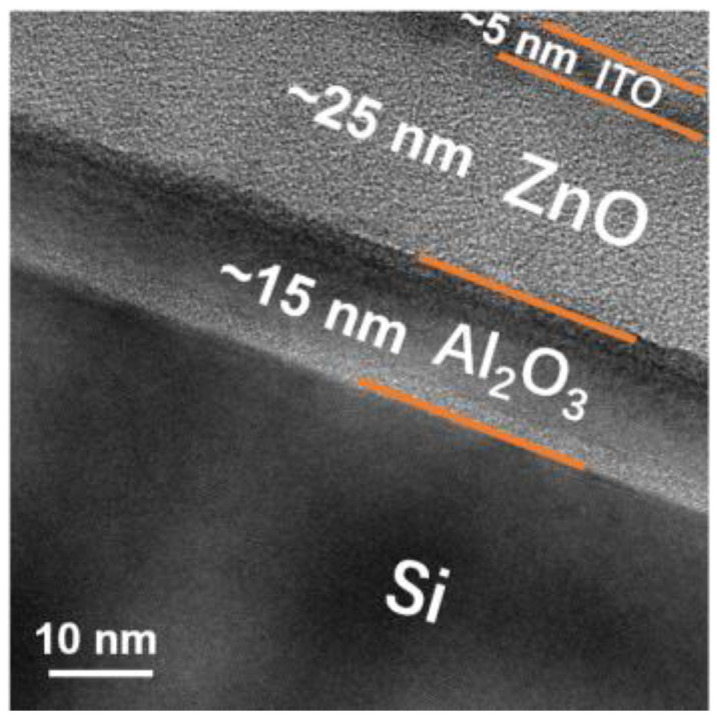
Cross-sectional TEM image of the Al_2_O_3_/ZnO/ITO film. Al_2_O_3_/ZnO/ITO film was deposited on Si substrate.

We conducted TEM measurement on the Al_2_O_3_/ZnO/ITO film, as shown in Figure 5. The figure shows that the Al_2_O_3_, ZnO, and ITO layers were stacked in order on Si substrate, and their respective thicknesses were about 15, 25, and 5 nm.

**Figure 6:**
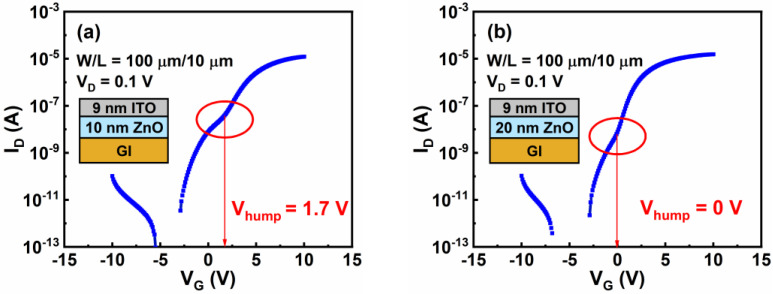

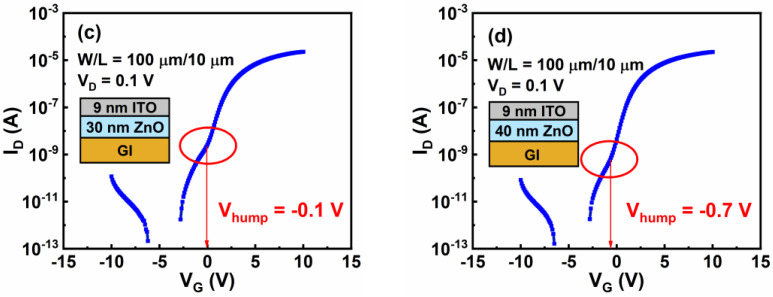
I_D_–V_G_ curves for group A TFTs. The t_ZnO_ values are (**a**) 10 nm, (**b**) 20 nm, (**c**) 30 nm, and (**d**) 40 nm. The t_ITO_ value is 9 nm.

To investigate the effects of the thickness of the ZnO layer (t_ZnO_) on the hump characteristics, drain current–gate voltage (I_D_–V_G_) curves of the group A TFTs were measured, as shown in Figure 6a–d. For the sake of brevity, V_hump_, which is the gate voltage corresponding to occurrence of the hump, was marked by red ellipses. We found that V_hump_ negatively shifted from 1.7 to −0.7 V but V_ON_ remained nearly constant as t_ZnO_ increased from 10 to 40 nm. The parameters of the group A TFTs are listed in Table 2, including the field-effect mobility (µ_FE_), V_hump_, and V_ON_.

**Figure 7:**
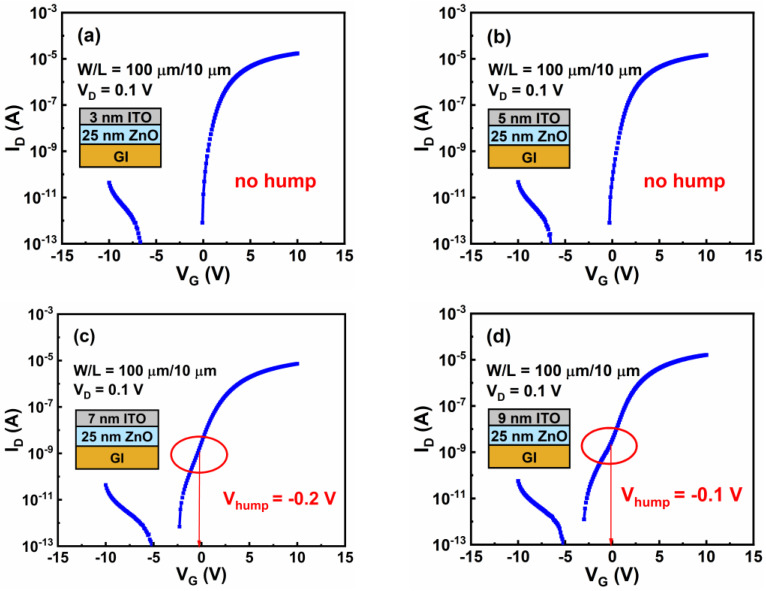
I_D_–V_G_ curves for group B TFTs. The t_ITO_ values are (**a**) 3 nm, (**b**) 5 nm, (**c**) 7 nm, and (**d**) 9 nm. The t_ZnO_ value is 25 nm.

To explore the effects of the thickness of the ITO layer (t_ITO_) on the hump characteristics, the I_D_–V_G_ curves of the group B TFTs were also measured, as shown in Figure 7a–d. We can see that V_ON_ negatively shifted from −0.1 to −3 V as t_ITO_ increased from 3 to 9 nm. Clearly, TFTs with both 3-nm and 5-nm ITO layers showed no hump, so we defined their V_hump_ to be equal to V_ON_ values of −0.1 and −0.3 V, respectively. TFTs with 7-nm and 9-nm ITO layers, respectively, were found to have a V_hump_ of −0.2 V and −0.1 V. The parameters of the group B TFTs are also listed in Table 2. Based on the above results, it could be determined that V_hump_ depended on t_ZnO_, while V_ON_ and μ_FE_ mainly depended on t_ITO_.

**Figure 8:**
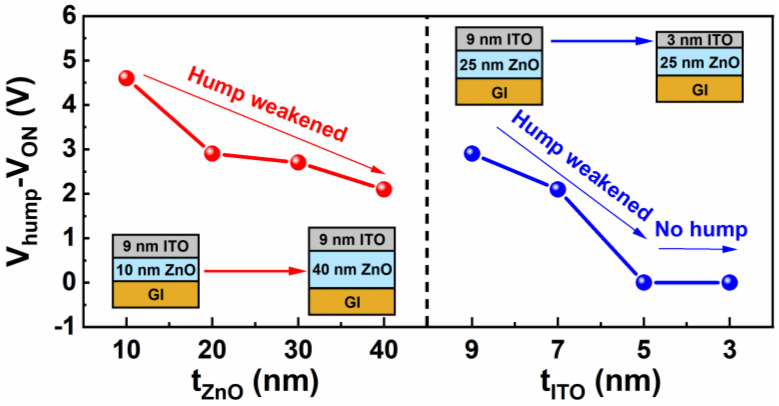
V_hump_–V_ON_ versus t_ZnO_ and t_ITO_.

The value of V_hump_–V_ON_ as a function of t_ZnO_ and t_ITO_ is depicted in Figure 8. We found that V_hump_–V_ON_, which is defined as an important parameter to measure the severity of hump phenomena, was negatively correlated with t_ZnO_, but was positively correlated with t_ITO_. Notably, by optimizing t_ITO_ ≤ 5 nm, the hump could be eliminated.

According to previous studies in the literature, the existence of two or more current paths is one of the important reasons leading to hump formation [12,16,18,21,29]. In this work, the generation of hump phenomena in ZnO/ITO TFTs should have resulted from the dual current paths through both the ZnO and ITO layers. Based on the experimental results, namely that V_hump_ depended on t_ZnO_ and V_ON_ depended on t_ITO_, we could determine that the ITO current path turns on before the ZnO current path, at V_GS_ = V_ON_, while the ZnO current path turns on at V_GS_ = V_hump_.

**Figure 9:**
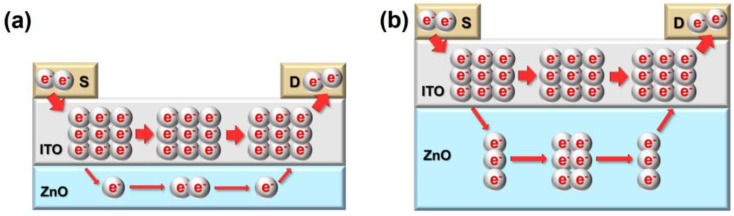

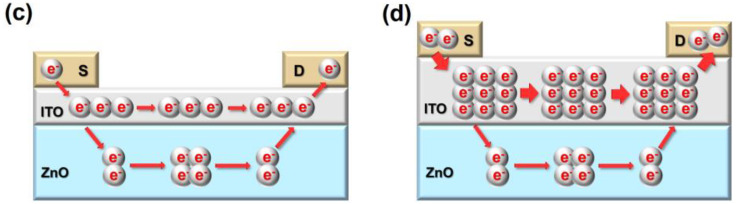
Schematic diagrams of current conduction in channel structures of (**a**) ZnO/ITO (10 nm/9 nm), (**b**) ZnO/ITO (40 nm/9 nm), (**c**) ZnO (25 nm)/ITO (≤5 nm), and (**d**) ZnO (25 nm)/ITO (>5 nm).

To provide the underlying mechanism of the hump characteristics of the ZnO/ITO TFTs, schematic diagrams that describe the current conduction in different channel structures are depicted, as shown in Figure 9a–d. For experimental group A, as t_ZnO_ increased from 10 to 40 nm, the carrier number in the ZnO layer increased and the V_hump_ was negatively shifted; thus, V_hump_–V_ON_ presented a decreasing trend, and the hump was weakened. Nevertheless, as the total current was mainly affected by the ITO layer with high carrier concentration, the total current remained nearly constant and resulted in a stable μ_FE_. For experimental group B, the current flowing through the ITO layer was improved as t_ITO_ increased, as shown in Figure 9c,d. Thereby, as t_ITO_ increased, V_ON_ was negatively shifted, μ_FE_ and V_hump_–V_ON_ presented a positive correlation with t_ITO_, and the hump was strengthened. Based on these results, it is clear that methods aimed at reducing the carrier concentration of the top channel layer—For example, adopting suitable post-processing treatment and optimizing process parameters—can be applied to suppress hump phenomena, and thus, these methods need further investigation.

**Figure 10:**
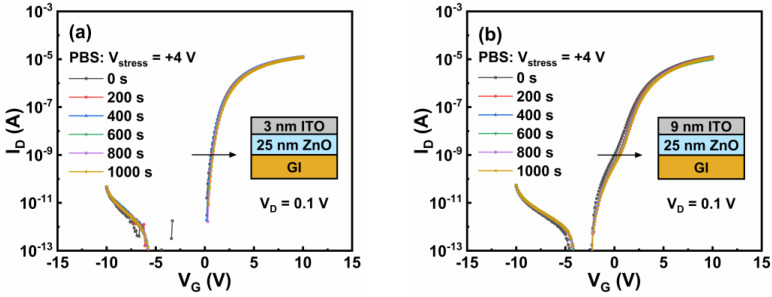
PBS stability for ZnO/ITO TFTs with t_ITO_ values of (**a**) 3 and (**b**) 9 nm. The t_ZnO_ is 25 nm.

**Figure 11:**
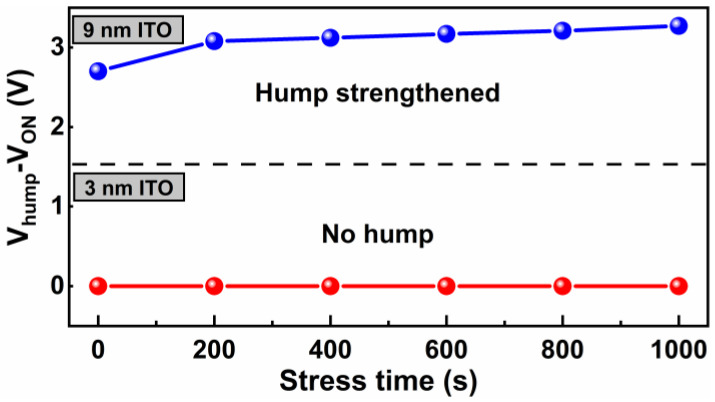
V_hump_–V_ON_ versus PBS time for ZnO/ITO TFTs with t_ITO_ values of 3 and 9 nm. The t_ZnO_ value is 25 nm.

Considering that many reported hump phenomena are induced by PBS, the PBS dependence of the hump characteristics was also investigated. Figure 10 displays the effects of PBS on the transfer characteristics of group B TFTs with t_ITO_ of 3 and 9 nm, and the value of V_hump_–V_ON_ plotted against stress time is shown in Figure 11. With increasing stress time, no hump was created for TFTs with t_ITO_ values of 3 nm, but the hump was strengthened for TFTs with t_ITO_ values of 9 nm. Under PBS, electrons were trapped in Al_2_O_3_ dielectric or/and at the Al_2_O_3_/ZnO interface, which influenced the shielding gate electric field [30,31]. When the t_ITO_ was 3 nm, V_ON_ and V_hump_ increased simultaneously with the stress time, so no hump occurred. When the t_ITO_ was 9 nm, V_hump_ increased but V_ON_ remained constant with stress time, and this was a result of the high carrier concentration of the ITO layer.

## 4. Conclusions

In conclusion, we first studied the material properties of the transparent conductive oxides ZnO and ITO. Further, we fabricated ZnO/ITO heterojunction TFTs and researched the effects of the channel structure on the hump characteristics. Our results showed that V_hump_–V_ON_ negatively correlated with t_ZnO_ but positively correlated with t_ITO_. In particular, the hump was eliminated when the t_ITO_ did not exceed 5 nm. Based on these results, current conduction in two channel layers is proposed to explain these hump characteristics. It was also found that the devices showed more severe humps under PBS for thicker ITO layers. This study extends the practical application of TCO and is helpful in terms of overcome the hump phenomenon of the oxide TFTs.

## Figures and Tables

**Table 1:** Hall-effect results of ZnO and ITO films. ZnO and ITO films were deposited on glass substrates.

Sample	Carrier Concentration (cm^−3^)	Conductivity (Ω^−1^cm^−1^)
ZnO	1.63 × 10^16^	1.78 × 10^−2^
ITO	2.80 × 10^19^	78.91

**Table 2:** Electrical parameters of ZnO/ITO TFTs, including µ_FE_, V_hump_, and V_ON_.

	**t_ZnO_ (nm)**	**µ_FE_ (cm^2^/Vs)**	**V_hump_ (V)**	**V_ON_ (V)**
**Group A**	10	10.71	1.7	−2.9
	20	11.28	0	−2.9
	30	12.55	−0.1	−2.8
	40	12.31	−0.7	−2.8
	**t_ITO_ (nm)**	**µ_FE_ (cm^2^/Vs)**	**V_hump_ (V)**	**V_ON_ (V)**
**Group B**	3	13.06	−0.1	−0.1
	5	15.69	−0.3	−0.3
	7	18.30	−0.2	−2.3
	9	18.64	−0.1	−3.0

## Data Availability

The data that support the findings of this study are available from the corresponding authors upon reasonable request.

## References

[1] Fortunato E., Barquinha P., Martins R. (2012). Oxide semiconductor thin-film transistors: A review of recent advances. Adv. Mater..

[2] Shi J., Zhang J., Yang L., Qu M., Qi D.C., Zhang K.H.L. (2021). Wide bandgap oxide semiconductors: From materials physics to optoelectronic devices. Adv. Mater..

[3] Samanta S., Chand U., Xu S., Han K., Wu Y., Wang C., Kumar A., Velluri H., Li Y., Fong X. (2020). Low subthreshold swing and high mobility amorphous Indium-Gallium-Zinc-Oxide thin-film transistor with thin HfO_2_ gate dielectric and excellent uniformity. IEEE Electron Device Lett..

[4] Lee S.Y. (2020). Comprehensive review on amorphous oxide semiconductor thin film transistor. Trans. Electr. Electron. Mater..

[5] Li H., Han D., Yi Z., Dong J., Zhang S., Zhang X., Wang Y. (2019). High-performance ZnO thin-film transistors prepared by atomic layer deposition. IEEE Trans. Electron Devices.

[6] Li Q., Dong J., Han D., Wang Y. (2021). Effects of channel thickness on electrical performance and stability of high-performance InSnO thin-film transistors. Membranes.

[7] Park J.C., Lee H.N. (2012). Improvement of the performance and stability of oxide semiconductor thin-film transistors using double-stacked active layers. IEEE Electron Device Lett..

[8] Kim J.I., Ji K.H., Jung H.Y., Park S.Y., Choi R., Jang M., Yang H., Kim D.H., Bae J.U., Kim C.D. (2011). Improvement in both mobility and bias stability of ZnSnO transistors by inserting ultra thin InSnO layer at the gate insulator/channel Interface. Appl. Phys. Lett..

[9] Furuta M., Koretomo D., Magari Y., Aman S.G.M., Higashi R., Hamada S. (2019). Heterojunction channel engineering to enhance performance and reliability of amorphous In–Ga–Zn–O thin-film transistors. Jpn. J. Appl. Phys..

[10] Yang J., Liao P.Y., Chang T.C., Chen B.W., Huang H.C., Chiang H.C., Su W.C., Zhang Q. (2017). Investigation of a hump phenomenon in back-channel-etched amorphous In-Ga-Zn-O thin-film transistors under negative bias stress. IEEE Electron Device Lett..

[11] Choi S.H., Han M.K. (2012). Effect of channel widths on negative shift of threshold voltage, including stress-induced hump phenomenon in InGaZnO thin-film transistors under high-gate and drain bias stress. Appl. Phys. Lett..

[12] Tsai Y.S., Chen J.Z. (2012). Positive gate-bias temperature stability of RF-sputtered Mg_0.05_Zn_0.95_O active-layer thin-film transistors. IEEE Trans. Electron Devices.

[13] Lee J.H., Ahn C.H., Hwang S., Woo C.H., Park J.-S., Cho H.K., Lee J.Y. (2011). Role of the crystallinity of ZnO films in the electrical properties of bottom-gate thin film transistors. Thin Solid Films.

[14] Lee J.Y., Lee S.Y. (2020). Investigation on hump mechanism in amorphous SiZnSnO thin-film transistor depending on Si concentration. Phys. Status Solidi A.

[15] Valletta A., Gaucci P., Mariucci L., Fortunato G., Templier F. (2008). “Hump” characteristics and edge effects in polysilicon thin film transistors. J. Appl. Phys..

[16] Teng T., Hu C.F., Qu X.P., Wang M. (2020). Investigation of the anomalous hump phenomenon in amorphous InGaZnO thin-film transistors. Solid State Electron..

[17] Chen H.C., Chen J.J., Tu Y.F., Zhou K.J., Kuo C.W., Su W.C., Hung Y.H., Shih Y.S., Huang H.C., Tsai T.M. (2020). Abnormal hump effect induced by hydrogen diffusion during self-heating stress in top-gate amorphous InGaZnO TFTs. IEEE Trans. Electron Devices.

[18] Kim W.S., Cho Y.J., Lee Y.H., Park J., Kim G., Kim O. (2017). Abnormal behavior with hump characteristics in current stressed a-InGaZnO thin film transistors. Solid State Electron..

[19] Hsieh S., Liang H.Y., Lin C.J., King Y.C. (2007). Stress-induced width-dependent degradation of low-temperature polycrystalline silicon thin-film transistor. Appl. Phys. Lett..

[20] Kim Y.M., Jeong K.S., Yun H.J., Yang S.D., Lee S.Y., Lee H.D., Lee G.W. (2012). Electrical instabilities in amorphous InGaZnO thin film transistors with Si_3_N_4_ and Si_3_N_4_/Al_2_O_3_ Gate Dielectrics. Jpn. J. Appl. Phys..

[21] Maeng W.J., Park J.S., Kim H.S., Kim E.S., Son K.S., Kim T.S., Ryu M., Lee S. (2011). The effect of active-layer thickness and back-channel conductivity on the subthreshold transfer characteristics of Hf-In-Zn-O TFTs. IEEE Electron Device Lett..

[22] Zhao M., Zhang Z., Xu Y., Xu D., Zhang J., Huang Z. (2020). High-performance back-channel-etched thin-film transistors with an InGaO/InZnO stacked channel. Phys. Status Solidi A.

[23] Zhao K., Xie J., Zhao Y., Han D., Wang Y., Liu B., Dong J. (2022). Investigation on transparent, conductive ZnO: Al films deposited by atomic layer deposition process. Nanomaterials.

[24] Barreca D., Garon S., Tondello E., Zanella P. (2000). SnO_2_ nanocrystalline thin films by XPS. Surf. Sci. Spectra.

[25] Jung H.Y., Kang Y., Hwang A.Y., Lee C.K., Han S., Kim D.-H., Bae J.-U., Shin W.-S., Jeong J.K. (2014). Origin of the improved mobility and photo-bias stability in a double-channel metal oxide transistor. Sci. Rep..

[26] Saha J.K., Billah M.M., Jang J. (2021). Triple-stack ZnO/AlZnO/YZnO heterojunction oxide thin-film transistors by spray pyrolysis for high mobility and excellent stability. ACS Appl. Mater. Interfaces.

[27] Kamiya T., Nomura K., Hosono H. (2009). Origins of high mobility and low operation voltage of amorphous oxide TFTs: Electronic structure, electron transport, defects and doping. J. Disp. Technol..

[28] Choi K.H., Kim H.K. (2013). Correlation between Ti source/drain contact and performance of InGaZnO-based thin film transistors. Appl. Phys. Lett..

[29] Jeong K.S., Kim Y.M., Lee G.W. (2015). Origin of oxygen-induced abnormal hump in bottom-gated polycrystalline zinc oxide thin film transistors. ECS J. Solid State Sci. Technol..

[30] Choi S., Choi S.J., Kim D.H., Park S., Kim J., Seo Y., Shin H.J., Jeong Y.S., Bae J.U., Oh C.H. (2020). Positive bias stress instability of InGaZnO TFTs with self-aligned top-gate structure in the threshold-voltage compensated pixel. IEEE Electron Device Lett..

[31] Yang J., Liao P.Y., Chang T.C., Chen B.W., Huang H.C., Su W.C., Chiang H.C., Zhang Q. (2017). H_2_O adsorption on amorphous In-Ga-Zn-O thin-film transistors under negative bias stress. Appl. Phys. Lett..

